# Genetic and comparative mapping of *Lupinus luteus* L*.* highlight syntenic regions with major orthologous genes controlling anthracnose resistance and flowering time

**DOI:** 10.1038/s41598-020-76197-w

**Published:** 2020-11-05

**Authors:** Nicole Lichtin, Haroldo Salvo-Garrido, Bradley Till, Peter D. S. Caligari, Annally Rupayan, Fernando Westermeyer, Marcos Olivos

**Affiliations:** CGNA (Agriaquaculture Nutritional Genomic Center), Las Heras 350, Temuco, Chile

**Keywords:** Genetics, Molecular biology, Plant sciences

## Abstract

Anthracnose susceptibility and ill-adapted flowering time severely affect *Lupinus luteus* yield, which has high seed protein content, is excellent for sustainable agriculture, but requires genetic improvement to fulfil its potential. This study aimed to (1) develop a genetic map; (2) define collinearity and regions of synteny with *Lupinus angustifolius*; and (3) map QTLs/candidate genes for anthracnose resistant and flowering time. A few linkage groups/genomic regions tended to be associated with segregation distortion, but did not affect the map. The developed map showed collinearity, and syntenic regions with *L. angustifolius*. Major QTLs were mapped in syntenic regions. Alleles from the wild parent and cultivar, explained 75% of the phenotypic variance for anthracnose resistance and 83% for early flowering, respectively. Marker sequences flanking the QTLs showed high homology with the *Lanr1* gene and *Flowering-locus-T* of *L. angustifolius*. This suggests orthologous genes for both traits in the *L. luteus* genome. The findings are remarkable, revealing the potential to combine early flowering/anthracnose resistant in fulfilling yield capacity in *L. luteus*, and can be a major strategy in the genetic improvement and usage of this species for sustainable protein production. Allele sequences and PCR-marker tagging of these genes are being applied in marker assisted selection.

## Introduction

Food security, soil fertility and sustainable food production can be significantly improved by the greater use and improvement of various grain legumes^1^ and especially *Lupinus spp.,* which have been traditionally used in human and animal diets^2,3^. They contribute to the sustainability of cropping systems because of their low requirement for fertilizer and positive input to soil fertility. They achieve this via their efficient mobilization of soil phosphorus and their fixation of atmospheric nitrogen (through their symbiotic relationship with *Bradyrhizobium)*^4–6^. Lupins belong to the genus Lupinus, of the genistoids clade of the papilionoid legumes^7^. There are an estimated 267 species of lupin distributed around the Mediterranean region (¨Old World¨) and North and South America (¨New World¨)^8^. A number of lupin species have been used in cultivation; the major ¨Old World¨ cultivated species are *Lupinus albus, Lupinus angustifolius* and *Lupinus luteus*, while *Lupinus mutabilis* is the major “New World” species^9,10^. Based on their seed coat structure, the Old World lupins are divided into two groups; the smooth-seeded and rough-seeded lupins. The smooth-seeded lupins include at least two lineages, *Angustifolius*-*Luteus* and *Micranthus*-*Albus*^11^. Seed protein content varies between lupin species, with *L. luteus* (2n = 52) showing the highest values^12^. Its proteins allow the production of high-quality food and feed, its isolate has functional and physicochemical properties suitable for the health-food industry^13–15^, while in feed for the aquaculture sector, it is the most prominent species^16^. However, this species still needs to be further adapted in order to represent a realistic alternative in supplying the demand for plant protein. This is made more complex under factors of climate change that affect many aspects of agricultural systems, including; temperature, water availability, change in pathogen spread, flowering time and host susceptibility to pests^17^. It is well documented that the “rusticity” of lupin species is a limitation to their expansion as a crop. Also lupin breeding has faced a new challenge in the last few decades, the lack of anthracnose resistance. Indeed, in *L. luteus*, no elite germplasm has been reported with resistance, despite it being accepted that one of the best sustainable practices to counteract diseases is the development and use of disease-resistant cultivars. It is true that some breeding lines, that show partial resistance to the disease have been identified^18^. Anthracnose resistance is critical to improve yield in lupin, the disease has become a serious problem worldwide, causing significant yield losses, as high as 100%, and a major limiting factor for lupin production. It is caused by *Colletotrichum lupini* (Bondar)^19,20^.

Another essential trait in the crop adaptation processes of *L. luteus* is flowering time, where it is important to breed germplasm suited to specific environmental conditions, thus directly improving yield. This trait is cited as an example of the consequence of bottlenecks, where a reduction in genetic variation for flowering time has previously occurred during domestication process^21^. Species like *L. luteus*, with its recent history of breeding, is likely to have a greater lack of diversity because its domestication has been based on a small number of founding individuals, and subsequent strong and persistent selection for key traits, all of which have resulted in severe genetic bottlenecks. Thus, the adaptation of *L. luteus* now needs to take this into account. In addition, the deficiency of information on which molecular and genetic is enormous compared to many other crops. Even though some work has been undertaken on these aspects^22–24^, there are still many gaps which need to be filled to help the species improvement.

Having this in mind, our strategy has been the exploration of novel genetic variability in wild germplasm, using advanced genomics, developed with model plants and the reference genome from cultivated and related species. For instance, no gene or markers associated with anthracnose resistance have been reported, and only recently some flowering time QTLs have been identified in *L. luteus*^24^. Whereas, greater progress had been achieved in *L. angustifolius*, which has been proposed as a reference genome in comparative studies^25,26^. In this species, the cultivar Tanjil, has been widely used for breeding anthracnose resistance. Its resistance is controlled by a single dominant gene, *Lanr1,* mapped on linkage group (LG) NLL-11, and its sequence is localised in a single Scaffold_133^26,27^. In flowering time, a major gene (*Ku*) has been identified and mapped on LG NLL-10; which controls the vernalisation requirement^28,29^. Recent studies showed that this major *Ku* gene corresponds to the *FLOWERING LOCUS T* homologues (*LanFTc1*), and that a deletion in the promoter region of it is responsible for the loss of vernalisation requirement^25,30^.

Synteny has been reported between the reference genome of *L. angustifolius,* with *L. albus*, and other model legume species^26,29,31^, which highlights the possibility of synteny and collinearity with *L. luteus*, since both species have been reported to share the same lineage and clade^11^.

In order to add more molecular and genetic information to this species, in this study a first attempt was made to develop the genetic linkage map of the *L. luteus* genome, and to compare it with *L. angustifolius*. NextRAD genotyping (Nextera fragmentation with Restriction Associated Digestion) was used. This technology utilizes a Nextera (Illumina, Inc.) reaction to fragment genomic DNA and then amplification using modified primers which are complementary; therefore, only fragments containing this recognition site are amplified. The result is a randomized collection of DNA fragments that represents a sub-fraction of the tested genome^32,33^. Applications using NextRAD include studies of genetic diversity of the Andean lupin, *Lupinus mutabilis*^34^, and an insect^33,35^. This, together with the phenotypic exploration of novel genetic variability for anthracnose resistance and flowering time genes, was used to go forward. Thus, a large mapping population from a cross between a *L. luteus* wild accession with an elite variety was used to explore and dissect these relevant traits. The main goal of this study was to (1) develop the genetic linkage map of *L. luteus* using de novo genome assembly and NextRAD genotyping; (2) define collinearity and regions of synteny with the reference genome *L. angustifolius*; and (3) map QTLs/candidate genes for anthracnose resistant and flowering time, in order to identify important orthologous candidate gene for both traits: key genetics factors for further developing the production of sustainable plant protein.

## Materials and methods

### Plant materials and mapping population

To develop the *L. luteus* genetic linkage map, a mapping population of 188 F_2_ individuals was generated by crossing Alu*Prot*-CGNA with Core 98 (PI385149). The female parent, Alu*Prot*-CGNA (cultivar developed in Chile by CGNA and now grown in southern Chile) is a very early flowering variety^15^, with good yield but is susceptible to anthracnose. The male parent, the CGNA’s Core 98, is a wild accession, which has shown resistant to anthracnose and very late flowering time. The cross was made under greenhouse conditions (16-h light, 23 °C and 60–75% relative humidity). The F_1_ was grown and selfed to obtain the F_2_ mapping population of 188 individuals. In order to develop the *L. luteus* genetic linkage map, and to carry out QTL analysis, an F_2:3_ population was then developed by selfing each of the F_2_ individuals, generating 188 F_2:3_ families. Each family was then divided in two, generating two populations, one was used to evaluate flowering time (188 F_2:3_ families) and the other (100 F_2:3_ families) to evaluate anthracnose resistance. Both F_2:3_ populations were evaluated under field condition, in order to validate the F_2_ phenotypic data and QTLs for these traits.

### Isolation of genomic DNA and development of NextRAD markers

Young leaves were collected from each F_2_ individual of the mapping population and the two parental lines. DNA extraction was based on a modified CTAB protocol developed at CGNA, as described by Parra-Gonzalez et al.^22^. Extracted DNA was quantified using fluorometric quantification (Qubit) and diluted to 50 ng/μl. Genomic DNA was converted into NextRAD genotyping-by-sequencing libraries by SNPsaurus (SNPsaurus, LLC)^32^. Genomic DNA was first fragmented with Nextera reagent (Illumina, Inc), which also ligated short adapter sequences to the ends of the fragments. The Nextera reaction was scaled for fragmenting 20 ng of genomic DNA. Fragmented DNA was then amplified as follows: 26 cycles at 73 °C, with the primer matching the adapter and extending 9 nucleotides into the genomic DNA with the selective sequence GTGTAGAGC. Thus, only fragments starting with a sequence that hybridized with the selective sequence of the primer was efficiently amplified. The NextRAD libraries were sequenced on an Illumina HiSeq 4000 instrument with two lanes of 150 bp single-end reads (at the University of Oregon). Parental lines were sequenced separately to develop the short-read reference sequence to map the data from the F_2_ population. Thus, marker data can be collected without the need to produce a high quality reference genome. Bioinformatics analysis of data including SNP calling was provided by the NextRAD service provider using a reference-independent pipeline (SNPsaurus, LLC). Briefly, the reads were trimmed using bbduk (BBMap tools, https://sourceforge.net/projects/bbmap/) with the following parameters: bbmap/bbduk.sh in = out = ktrim = r k = 17 hdist = 1 mink = 8. The parental samples were used to identify a set of polymorphic loci and to generate a set of reference DNA sequences in the FASTA text-based format. The reads were then mapped to this FASTA file with an alignment identity threshold of 0.98, using bbmap. Genotype calling was achieved using call variants (BBMap tools). Genotype imputation was not used. A genotype table was provided for use with JoinMap (see below), along with a Variant Call Format (VCF) file of data from all samples, and a FASTA formatted file containing the short-read sequence common to both parents. These data were used to evaluate sequencing coverage of the sample. Sequencing depth (DP) at each marker for each sample was evaluated using DP values from the Variant Call Format (VCF) file that contained nucleotide variation predicted by the software. The heatmap of sequencing depth at each marker for each sample was created using DP values from the VCF file, using heatmap.bp in R package vcfR with zero values omitted (https://www.R-project.org/)^36^. Mean DPs for all F_2_ samples were plotted, using the barplot function in R.

### De novo genome assembly and PCR marker development

Additional molecular markers were developed using *L. luteus* scaffold sequences created from low-coverage whole genome sequencing of the parental line Core 98, by sequencing-by-synthesis using Illumina Hi-Seq 2000, followed by de novo assemblage, using SOAPdenovo2. Scaffold statistics were prepared using QUAST^37^. Fastq files in order to prepare scaffold sequences and raw sequence data used to create NextRAD markers, which are deposited in the NCBI Sequence Read Archive (BioProject accession PRJNA594652, https://www.ncbi.nlm.nih.gov/sra/PRJNA594652). The scaffold sequence was then subjected to BLAST with the sequences of some PCR markers already mapped on the *L. angustifolius* map^25,29^. The markers where those evenly distributed over the *L. angustifolius* map. Analysis of the DNA sequences between the species was carried out using Geneious v.6.1.8 software (https://www.geneious.com)^38^. Primers were designed using the algorithm Primer3. Amplifications were carried out in 20 ul PCR reactions containing 100 ng of genomic DNA, 0.2 mM dNTPs, 2 mM MgCl2, 1X PCR buffer and 2.5% DMSO (only for SSR (Simple sequence repeats) markers), 1U Taq polymerase (GoTaq G2, Promega) and 2.5 mM of each reverse and forward SSR primer; and 1 mM of each reverse and forward SNP primer. The PCR protocol was as follows: 95 °C for 5 min, 30 cycles of 1 min at 95 °C, 1 min at 58 °C, 1 min at 72 °C, and a final extension at 72 °C was performed for 5 min. SSR PCR products were separated on 6% denaturing polyacrylamide gels (urea 8 M) run in 1X TBE buffer at 60 watts for 3–4 h and their polymorphised products visualized using silver stain procedures^22,39^. SNP PCR products were separated on 8% polyacrylamide gels using Asymmetric Single Stranded Conformational Polymorphism technologies (SSCP)^40^, run in 1X TBE buffer at 2–3 watts for 16–18 h and polymorphism visualized using silver stain procedures. The mapping population was genotyped with polymorphic co-dominant PCR markers, identifying parental loci as homozygous (allele A or B), and the heterozygous loci constitution (allele H). The allelic constitution for each F_2_ individual was entered on to a matrix in Excel (Microsoft Corporation) for linkage analysis.

### Construction of genetic linkage map

Pairwise analysis, grouping of markers and mapping, were performed with JoinMap 4.0^41^. Single locus analysis (JoinMap Single Locus Analysis, JMSLA) was applied to detect possible markers or regions with segregation distortion. Since distorted markers were detected, two marker datasets were defined: one set contained all markers, and the other having markers without significant segregation distortion. Linkage analysis and mapping was carried out with both datasets. This was in order to establish the most accurate map and QTL analysis. The map was constructed based on recombination frequencies and LOD values. The markers were assigned to LGs based on (modified) LOD scores of pairs of markers. In the grouping of markers, LOD values from 1 to 8 were used to detect the stability of grouping. Markers in the group were analyzed for pairwise linkages with initial set up values of REC and LOD thresholds of 0.499 and 1, respectively. Strong linkage was considered to be present with a REC smaller than 0.01 or a LOD larger than 10. A map distance was calculated using Kosambi’s mapping function^42^.

### Comparative mapping of *L. luteus* and *L. angustifolius* along with physical location of markers

The 150 bp marker sequences from NextRAD sequencing along with PCR marker sequences were ordered according to the LGs prepared with JoinMap. These were used as queries against the *Lupinus angustifolius* reference genome (LupAngTanjil v1.0) in a local megablast search where the top scoring hits were retrieved (blastn-task megablast-query-db-outfmt 6-num_alignments 1-out). Chromosome name, start and end positions were extracted with awk. Synteny block size was calculated by counting the distance in base pairs between adjacent markers that mapped to the same *L. angustifolius* chromosome. Alignments of *L. luteus* markers to *L. angustifolius* unassigned scaffold sequences were not considered for this calculation. Circular plots were prepared using circos^43^, and lollipop chart using R package ggplot2^44^. The *L. angustifolius* genome build contains 13,564 scaffold sequences. For clarity of graphical representation, alignments to these regions were removed from the circos plots. Linear comparisons were prepared using starting base pair positions of BLAST hits on *L. angustifolius* chromosomes drawn using the R package LinkageMapView and manually connected to the *L. luteus* genetic map^45^. In order to define collinearity and marker order between *L. luteus* map and *L. angustifolius* map, common markers anchored in both maps were compared along LGs. The order and map interval were then calculated.

### Phenotypic evaluation and data analysis for anthracnose resistance and flowering time

The mapping population of 188 F_2_ individuals, together with the parents, were assessed by QTL analysis for flowering time and anthracnose resistance. Flowering time, measured as days to flowering (DTF), represents the period of time from sowing until the first whorl was fully open. The F_2_ population was evaluated for DTF under field conditions at Vilcún (Southern Chile, La Araucanía Region, 38° 41′ 44.04″ S and 72° 25′ 1.94″ O), over the winter growing season 2013–2014. The segregating population of 188 F_2:3_ families was evaluated for DTF in field conditions at Huichahue (Southern Chile, La Araucanía Region, 38° 50′ 6.30″ S and 72° 31′ 2.22″ O) over the winter growing season 2016–2017, using a completely randomized block experimental design with three replications. Each replication comprised 20 plants.

Anthracnose resistance was evaluated in in vitro conditions as describe by Cuccuza and Kao^46^ using the 188 F_2_ individuals of the mapping population. *Colletotrichum lupini* var. *setosum*^19^ was isolated from infected plants of cultivar Alu*Prot*-CGNA, collected from different locations in the southern region of Chile, a major area of lupin production. *Colletotrichum lupini* was used, since in southern Chile it has been widely reported as the only detected and causal agent for anthracnose in lupin^20^. All fungi collected showed cultural and morphological features of *Colletotrichum lupini* var. *setosum* as reported by Nirenberg et al.^19^. Infected plant organs were surface-sterilized (water for 20 min, 35% ethanol, 0.5% sodium hypochlorite for 1 min respectively and three washes with sterile distilled water for 1 min) and placed onto potato dextrose agar (PDA). Cultures were then grown on PDA at 25 °C until fungus growth was observed (3–5 days). Disks from the edge of the active colony growth were transferred aseptically to new Petri dishes with PDA media. These cultures were incubated under the same conditions for 7–10 days. Two cotyledons from each of the 188 F_2_ and parental lines were collected. The inoculation was performed with a conidial suspension of 1 × 10^6^ conidia per ml into an injury of 2 mm length on the upper surface of the cotyledon. The inoculated cotyledons were incubated in a moistened Petri dish at 23 ± 2 °C under 16-h photoperiod of white fluorescent light (2000 lx). The evaluation was carried out 10 days after inoculation. To evaluate the degree of damage, a scale from 1 to 5 was used. Cotyledons were given the following scores: Score 1 when they exhibited a spot of soft yellow color in the area of inoculation. Score 2, a spot of necrosis in the area of inoculation. Score 3, a little localized hypha (diameter less than 2 mm). Score 4, hyphae with diameter more than 4 mm. Score 5, abundant presence of fungus and tissue degradation. In order to validate the anthracnose resistance observed in the F_2_ mapping population under in vitro assays, 100 F_2:3_ families derived from the F_2_ mapping population were tested for anthracnose resistance under field conditions, as described by Fischer et al.^47^. A completely randomized block experimental design was used with three replications, where each replication was represented by 20 plants of each F_2:3_ family sown in two rows (10 plants per row planted 10 cm apart and 20 cm between rows), and one infection row of 10 plants (seed of the susceptible parent cv. Alu*Prot*-CGNA was inoculated with the suspension of strain *Colletotrichum. lupini,* as in vitro assay) in the middle, to obtain a high and permanent infection pressure. The resistant parent (Core 98) was also included as a control, and similarly to F_2:3_ families, was sowed with an infection row. Infection rows were sown two weeks after sowing the F_2:3_ families. The data were collected from all the plants in all the plots, scoring the resistant plants (non-infested) and susceptible plants (infested), as described by Fischer et al.^47^.

Analysis of variance (ANOVA) (SAS Institute, Cary, NC, USA) was carried out to explore the phenotypic variation of anthracnosis resistance and DTF in the F_2:3_ families. Estimates of the extent of genotypic and phenotypic variation calculated following Burton and DeVane^48^ as well as the broad sense heritability (H^2^)^49^. Treatment means were separated by Tukey test for P ≤ 0.05.

### Validation of major QTLs

When a major QTL, either for anthracnose resistance or DTF, was detected and mapped in the F_2_ population, markers previously reported as co-segregating for these traits in *L. angustifolius* and mapped in this study in *L. luteus* with highest LOD score, were chosen to validate the QTLs in the F_2:3_ families. Thus, tracing back the genetic classification of the F_2_ population at both loci and validating QTLs already mapped. Markers co-segregating with these traits were selected for further application in MAS.

### QTL analysis and mapping

DTF and anthracnose resistant segregation were evaluated in the F_2_ population, assuming the presence of one main locus and a Mendelian segregation of 3:1. Significant deviations were tested using the goodness of fit chi-square test (*X*^2^), where rejection of the 3:1 segregation was applied at P < 0.05. In the first instance an inspection for the presence of a QTL was carried out using a non-parametric approach, Kruskal–Wallis test, interval mapping (IM) was then applied. Both methods were implemented in MapQTL 6^50^. In IM LOD thresholds with significance level (P < 0.05) were empirically determined for each trait using the permutation test (1000 iterations). Once a single QTL was detected and mapped, in order to search for others minor segregating QTLs, further analysis was then carried out using Genome-wide Composite Interval Mapping (GCIM)^51^, implemented by the R package QTL.gCIMapping.GUI v2.0 (https://cran.r-project.org/web/packages/QTL.gCIMapping.GUI/index.html)^52^. Random model was used with a walk speed for genome-wide scanning of 1 cM, and the LOD score thresholds of 3 for significant QTLs.

To identified candidate genes in the QTL regions for anthracnose resistance and DTF, a search was carried out in the Lupin Genome Portal (https://www.lupinexpress.org), through BLAST in the nucleotide and protein database (Narrow-leafed lupin genome scaffold assembly v1.0, Narrow-leafed lupin annotated gene v1.0)^26^ and the NCBI database (https://www.ncbi.nlm.nih.gov/) was also used in order to confirm suggested candidate genes.

## Results

### Genetic linkage map construction

By using the NextRAD sequencing approach, 834 polymorphic sites were identified (Supplementary Table S1), which generated 685 high quality SNP markers. A heatmap analysis revealed parental lines with high coverage (Supplementary Fig. S1). Mean coverage in F_2_ individuals ranged from 0.15 to 155.08 (Supplementary Fig. S2, Supplementary Table S2). 12 F_2_ individuals with a high percentage of missing data were identified as assay failures and removed (Supplementary Fig. S1). The adjusted average mean coverage for all F_2_ individuals was 63.13. Mean per marker coverage ranged from 26.47 to 254.20 (Supplementary Table S2). Mean coverage for the parental mapping population, Alu*Prot*-CGNA and Core 98 was 495.44 and 433.65, respectively.

Markers developed from the de novo assembled *L. luteus* genome revealed 311,789 scaffolds ≥ 500 bps, with N50 = 2495 and L50 = 49,228 (Supplementary Table S3). The BLAST results for these scaffolds with genomic sequences of some PCR markers of *L. angustifolius* map generated 28 additional analogous PCR markers in the *L. luteus* genome (Supplementary Table S4).

**Figure 1:**
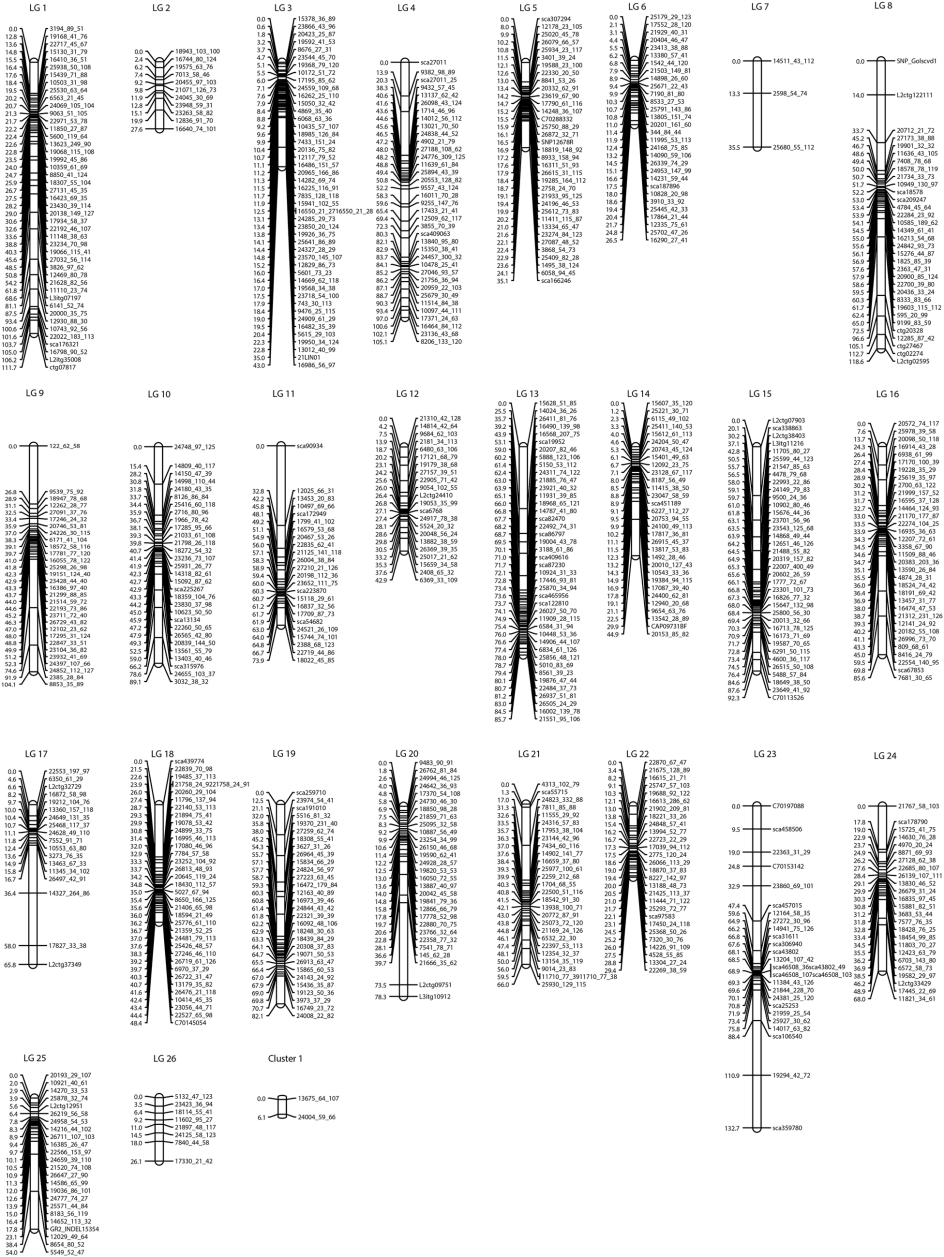
Genetic linkage map of *L. luteus* genome comprising 744 loci distributed among 26 linkage group (LG1–LG26).

A genetic linkage map was constructed with high LOD score (LOD = 6), which allowed detection of all markers with strong linkage, and stable marker order per LG. The map fell into 26 LGs as expected for the *L. luteus* genome, with a total map length of 1,772 cM and 744 loci with a mean density of one marker every 2.8 cM. The map had the largest gap on LG 20 (33.8 cM) and the smallest gap on LG 6 (3.1 cM) (Fig. 1, Table 1). The loci were identified with co-dominant markers, SNPs, SSR and INDEL. LG length ranged from 132.7 cM to 26.1 cM, with a mean of 68.2 cM. The highest number of loci mapped per LG was 47, while the lowest was 3, with a mean of 28.6 markers per LG (Table 1). Two markers, linked together, did not link to LGs and 0.9% of markers remained unlinked. LGs showed a cluster of markers and longer intervals in specific genomic regions, which is likely to reflect an uneven distribution of recombination frequencies along *L. luteus* chromosomes (Fig. 1).

**Table 1:** Description of basic characteristics for 26 linkage groups in F_*2*_ mapping population of *L. luteus*.

LG	No. of markers	Map length (cM)	Mean density (cM)	Maximum gap (cM)	No. of distorted markers		Total distortion markers	Map length* (cM)	Mean genetic distance (cM)	Maximum gap (cM)
					(0.001 < P < 0.05)	(P ≤ 0.001)				
1	45	111.7	2.5	12.8	2	1	3	109.1	2.6	12.9
2	11	27.6	2.5	7.7	0	0	0	27.6	2.5	7.7
3	47	43.0	0.9	12.2	2	1	3	43.0	1.0	12.5
4	37	105.1	2.8	18.0	4	2	6	55.2	1.8	1.7
5	35	35.1	1.0	11.0	5	2	7	34.4	1.2	10.7
6	30	26.5	0.9	3.1	0	0	0	26.5	0.9	3.1
7	3	35.5	11.8	22.2	0	0	0	35.5	11.8	22.2
8	33	118.6	3.6	24.1	3	2	5	117.9	4.2	24.2
9	30	104.1	3.5	26.8	2	0	2	25.5	0.9	26.8
10	30	89.1	3.0	15.4	1	0	1	88.9	3.1	15.3
11	24	73.9	3.1	32.8	1	1	2	66.7	3.0	32.8
12	22	42.9	2.0	6.4	1	1	2	42.3	2.1	10.4
13	42	85.7	2.0	25.5	7	0	7	84.3	2.4	25.5
14	31	44.9	1.4	15.0	2	1	3	49.0	1.8	19.7
15	37	92.3	2.5	20.1	8	4	12	90.7	3.6	20.1
16	34	85.6	2.5	15.8	3	0	3	79.8	2.6	15.9
17	18	65.8	3.7	21.6	1	15	16	7.8	3.9	7.8
18	36	48.4	1.3	21.5	8	1	9	46.7	1.7	21.7
19	30	82.1	2.7	12.5	0	1	1	70.9	2.4	12.5
20	29	78.3	2.7	33.8	1	3	4	76.2	3.0	33.8
21	28	66.0	2.4	15.7	0	0	0	66.0	2.4	15.7
22	28	29.4	1.1	4.8	1	1	2	26.4	1.0	5.0
23	27	132.7	4.9	22.5	0	1	1	132.5	5.1	22.5
24	25	68.0	2.7	19.1	0	4	4	67.1	3.2	20.8
25	24	54.0	2.3	15.6	0	0	0	54.0	2.3	15.6
26	8	26.1	3.3	8.1	2	3	5	17.5	5.8	13.6
Total	744	1772.4	2.8		54	44	98	1541.5	2.9	
	100%				7.3%	5.9%	13.2%	87.0%		

*LG* Linkage group; *Map length (cM) without distorted markers.

### Effect of marker segregation distortion on map construction

Single locus analysis detected 5.9% of markers with highly significant (P* ≤ *0.001) distortion, and 7.3% with significant (0.001 < P < 0.05) segregation distortion, giving a total of 98 markers with distortion from the expected Mendelian segregation (Table 1). Among LGs, 61.5% had a maximum of 9.7% of markers with segregation distortion; LG2, LG6, LG7, LG21 and LG25 did not have distorted markers, and LG10, LG19 and LG23 had one distorted marker. LG17 (88.9% of markers with segregation distortion), LG26 (62.5%), LG15 (32.4%), LG18 (25%), LG5 (20%), LG13 (16.7%), LG4 (16.2%), LG24 (16%), LG8 (15.2%) and LG20 (13.8%) had the major proportion of markers with segregation distortion (Table 1, Supplementary Table S5). To see whether or not these distorted markers affected the map, a second run for linkage analysis and mapping using a marker dataset that excluded the distorted markers was carried out. As expected, 26 LGs were again generated, and map coverage was reduced by 13% (231 cM). However, LG17, LG9, LG4 and LG26 reduced their map length by 88.1%, 75.5%, 47.5% and 33%, respectively, and compromised 46% of the total map length reduction. LG17 was severely affected by removing distorted markers, it was almost unmapped, since just the minimum number of markers remained to establish linkage. This LG had the largest distorted genomic region (30 cM) with a cluster of markers (16 out of 18), with very significant (P ≤ 0.001) segregation distortion. This distortion was found to share a common feature, all allele frequencies of the 16 loci in the 188 F_2_ individuals were skewed toward homozygosity of the female allele (mean frequency of 42.7%), and only a mean frequency of 13.2% of homozygotes with male alleles, (Supplementary Table S6). The large map length reduction of LG9 is due to only two distorted markers. LG3 showed no reduction in its map length, and LG8, LG10 and LG23, their map lengths were reduced by 0.6%, 0.2% and 0.2%, respectively. LG24, despite 16% of its markers being removed, was almost unaffected in map length (1.3% of reduction). The largest map gap had minor changes (equal to an overall mean of 17 cM) and mean map genetic distance was only reduced in 0.1 cM. Marker order was almost unaffected, and those collinear markers and syntenic regions with *L. angustifolius* map and reference genome, presented equivalent map positions and order with respect to the map, including all markers (see below). Thus, the map involving all markers, was used for the further analysis of comparative mapping and QTL analysis.

### Comparative analysis of *L. luteus* with map and reference genome of *L. angustifolius*

**Figure 2:**
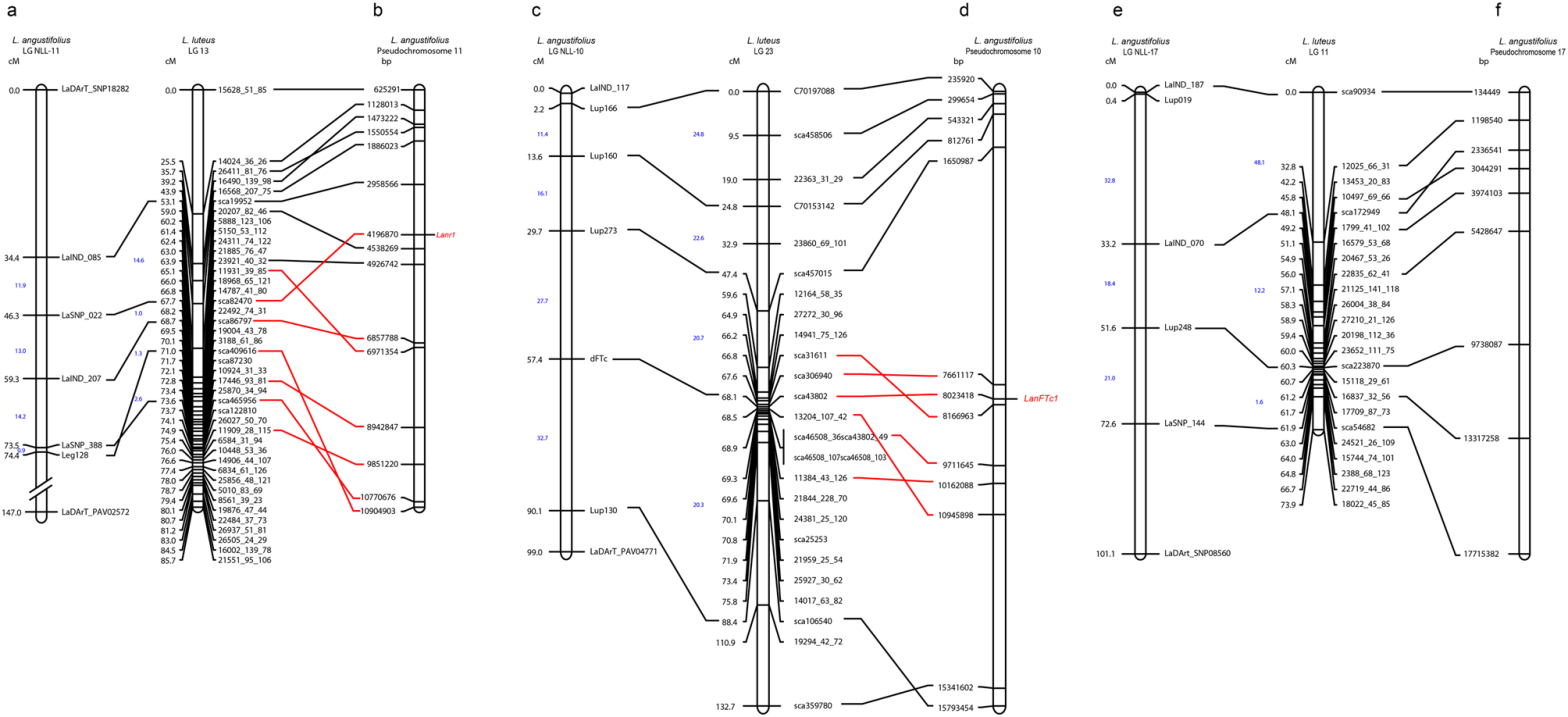
Comparative mapping and physical position of marker sequences of LGs of *L. luteus* in pseudochromosome (Chr) of *L. angustifolius*^26^. (**a**,**c**,**e**) collinearity between LGs of both lupin species. (**b**,**d**,**f**) Syntenic genomic regions with pseudochromosome of *L. angustifolius* in black lines, and in red lines regions containing genes controlling anthracnose resistance and DTF.

Comparing the *L. luteus* map with the referential consensus *L. angustifolius* map, which has been highly saturated, represented about 72% of the total map length, which is interesting considering that the loci mapped in *L. luteus* genome represents about 25.4% of the loci mapped on the consensus map of *L. angustifolius*. Markers already mapped in *L. angustifolius* were mapped onto the *L. luteus* map, representing 3.1% of loci, where LG11, LG13 and LG23, have 4, 5 and 5 common loci, respectively. Collinearity was observed between both species, i.e. *L. luteus* LG13, LG23 and LG11, were collinear with LG11, LG10 and LG17 of the *L. angustifolius* reference genetic map, respectively (Fig. 2a,c,e). Markers identified loci in similar genetic positions, in the same order, but with different map length intervals. Clearly *L. luteus* LGs had longer intervals map than *L. angustifolius* in one distal genomic region, with shorter intervals length at the other end. However, LG13 and LG11 of *L. luteus* which compare with LG11 and LG17 of *L. angustifolius*, respectively (Fig. 2a,e), exhibit equal cluster features of markers at one end of both LGs, which might indicate that both could share unequal chromosome arm lengths, indicating possible submetacentric chromosomes. Moreover, LG23 of *L. luteus* shared the same marker distribution with LG10 of *L. angustifolius*, with a clustering of markers in the central region (Fig. 2c), which could suggest similar metacentric features in this chromosome.

**Figure 3:**
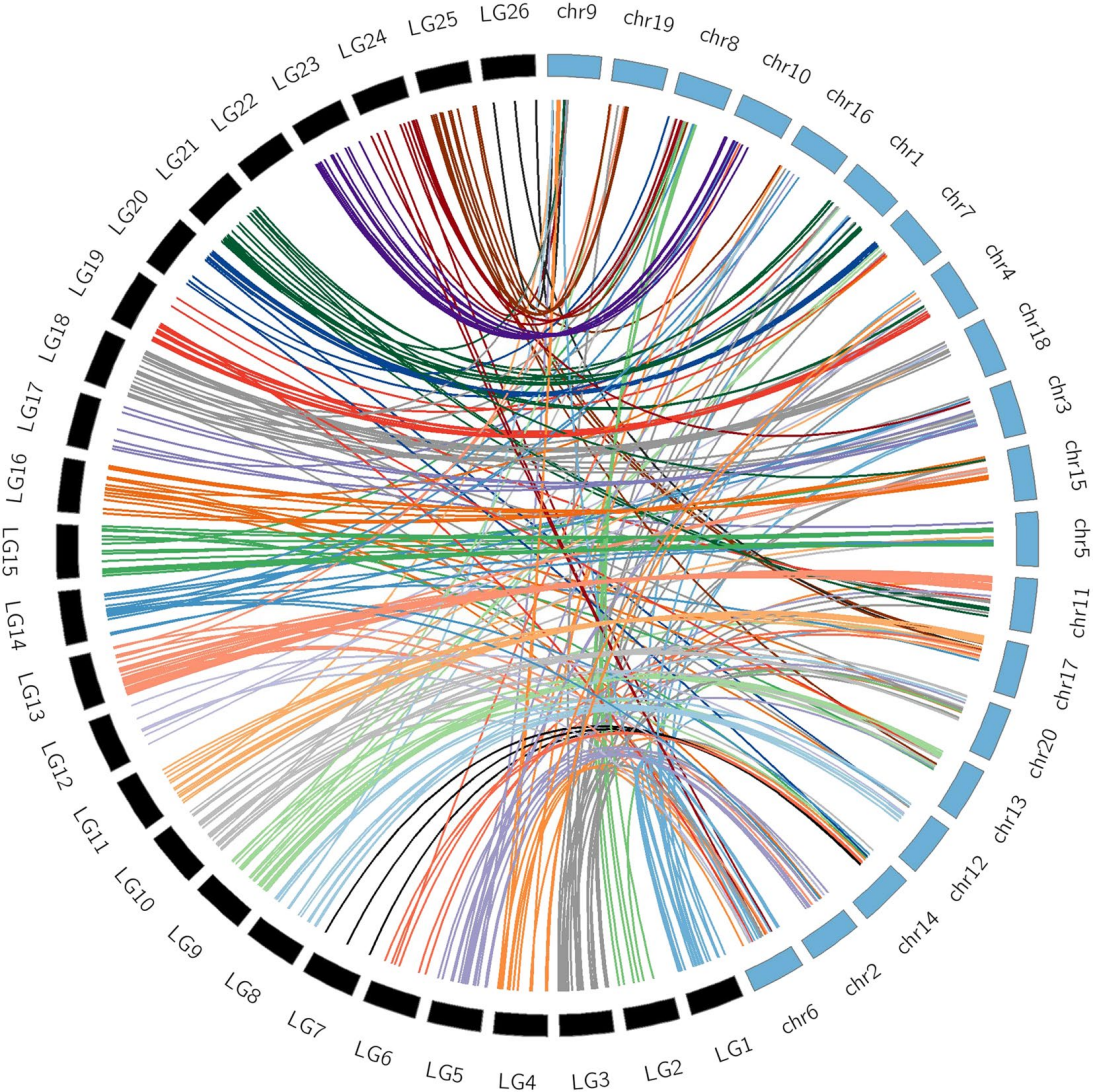
Syntenic link between linkage group (LG) of *L. luteus* and pseudochromosomes (Chr) of *L. angustifolius*. Pseudochromosomes and linkage groups are not drawn to scale.

A comparative analysis of marker sequences was performed with the *L. angustifolius* genome. In total, 56.7% of the marker sequences in *L luteus* produced homologous alignments with *L. angustifolius* pseudochromosomes. Of these, 28% aligned to scaffold sequences of *L. angustifolius.* Considering this sequence homology, *L. luteus* LG would correspond to the following *L. angustifolius* pseudochromosomes (with their relevant proportions): LG1-Chr6 (37.5%), LG2-Chr8 (62.5%), LG3-Chr1(14.3%), LG4-Chr6 (15.8%), LG4-Chr4 (15.8), LG5-Chr2 (43.8%), LG6-Chr14 (20%), LG6-Chr17 (20%), LG7-Chr14 (100%), LG8-Chr12 (57.1%), LG9-Chr13 (45%), LG10-Chr20 (38.9%), LG11-Chr17 (50%), LG13-Chr11 (53.6%), LG14-Chr3 (20%), LG15-Chr5 (58.8%), LG16-Chr15 (30.4%), LG17-Chr3 (36.4%), LG18-Chr18 (30.8%), LG19-Chr4 (33.3%), LG20-Chr7 (31.3%), LG21-Chr1 (43.8%), LG22-Chr16 (31.3%), LG23-Chr10 (60%), LG24-Chr8 (44.4%), LG25-Chr19 (26.7%) and, LG26-Chr9 (40%) (Fig. 3, Supplementary Fig. S3, Supplementary Tables S7, S8). Within *L. luteus* LG, LG13 showed two syntenic regions, a short one: 2315179 bp, and a larger one: 7775501 bp (Fig. 2b). LG23 gave the largest syntenic region with 15558191 bp (Fig. 2c). LG11 showed two syntenic regions, a short one: 5294198 bp, and a larger one: 7978928 bp (Fig. 2f). LG1 and LG4; LG4 and LG19, shared syntenic region with Chr6 and Chr4 of *L. angustifolius*, respectively (Supplementary Tables S7, S8).

### Mapping QTLs for anthracnose resistance and DTF in *L. luteus*

Clear and contrasting phenotypes for anthracnose resistance and DTF were observed in the parents and mapping population, allowing unambiguous phenotyping of the F_2_ mapping population. The mean frequency distribution for anthracnose resistance and DTF in the 188 F_2_ individuals followed a typical 3:1 Mendelian segregation (*X*^2^ = 0.1134, P = 0.75; *X*^2^ = 0.2553, P = 0.75 respectively, Supplementary Fig. S4), i.e. three classes of resistance level and flowering time, thus, as would be expected, they did not show normal distributions, as typifying the presence of a single dominant genes for anthracnose resistance and early flowering (Supplementary Fig. S4). The mean performance of the F_2:3_ population for anthracnose resistance, and the mean performance of the F_2:3_ population for DTF, evaluated under field condition, gave highly significant (P < 0.05) differences between families, and no significant effect due to replications (Table 2). Looking at each F_2:3_ family of each population for each trait, three phenotypic classes were observed, either for anthracnosis resistance or DTF: full resistance, ¾ resistance or fully susceptible and early flowering, ¾ early flowering or late type, which validated the genetic constitution of the loci under study as inferred from the F_2_, and the allelic dominance displayed in the next generation. High values of genotypic and phenotypic variances were observed for each trait. Broad sense heritabilities (H^2^) were obtained, and considering that H^2^ captures the proportion of the total variance due genetic effect, the high heritability values obtained confirm the strong genetic effect determining each trait (Table 2), which is in agreement with the result obtained from the F_2_ mapping population.

**Table 2:** ANOVAs for differences in anthracnose resistance and DTF, along with population parameters of the F_2:3_ families.

Source of variation	df	SS	MS	F	P	σ^2^g	σ^2^p	H^2^
**Anthracnose resistance**						58.1	58.9	0.98
F_2:3_ families	99	17,395.0	175.7	217.1	0.0000			
Replication	2	0.4	0.2	0.2	0.79			
Error	198	160.3	0.8					
**DTF**						31.6	35.8	0.88
F_2:3_ families	187	18,540.0	99.1	23.6	0.0000			
Replication	2	4.4	2.2	0.5	0.59			
Error	374	1574.0	4.2					

*df* degrees of freedom, *SS* sum of square, *MS* mean square, *σ*^*2*^*g* genotypic variance, *σ*^*2*^*p* phenotypic variance, *H*^*2*^ heritability.

**Figure 4:**
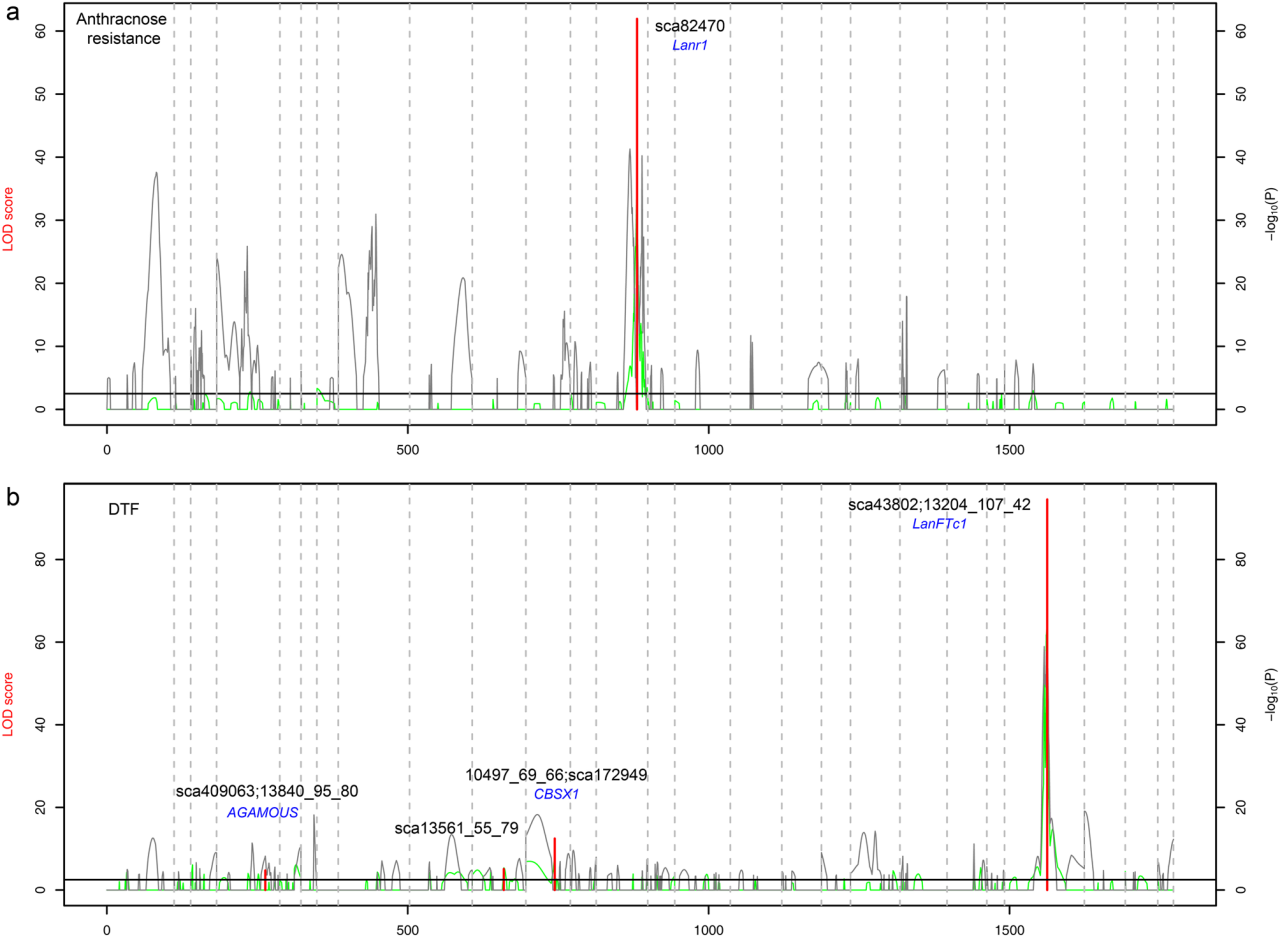
QTLs for anthracnose resistance (**a**) and DTF (**b**) in the F_2_ mapping population of *L. luteus* using QTL.gCIMapping.GUI. The LOD score of QTLs are marked in red line, and − log_10_(*P* value) for additive and dominance, are marked in grey and green lines, respectively. Black solid horizontal line is the LOD thresholds for significant QTLs. Candidate genes and flanking markers are shown by blue and black colors, respectively.

A major QTL for anthracnose resistance was identified and mapped in the *L. luteus* LG13 in the F_2_ population (Figs. 2, 4a). The non-parametric, Kruskal–Wallis test, revealed that markers mapped between 67.7 and 73.3 cM of LG13, were highly significantly associated with the phenotypic variation of the trait (P < 0.0001) (Supplementary Table S9). Interval mapping confirmed this result; a significant QTL was mapped in the same genomic region, and the marker sca82470, which mapped to a position of 67.7 cM, gave the highest LOD score (LOD = 56.5) in F_2_ (Table 3). As expected, the alleles from the wild, resistant parent at this locus explained 75% of the phenotypic variance of the trait in the F_2_ population (Table 3). Similarly, a single major QTL was also identified for DTF. The markers localized between 68.1 cM to 69.6 cM of LG23 were highly significantly associated with the variation in DTF (P < 0.0001) by Kruskal–Wallis test (Supplementary Table S9). Interval mapping detected a single major QTL, where marker sca43802, mapped at the position of 68.1 cM, presented the highest LOD score (LOD = 68.4) in the F_2_ population and explained 81.4% of the phenotypic variance for DTF (Fig. 2, Table 3). In both genomic regions neither clusters or distorted markers were mapped, thus giving greater confidence in the QTL positions.

**Table 3:** Anthracnose resistance and DTF QTLs in F_2_ and F_2:3_ population of *L. luteus*, detected by interval mapping and genome-wide composite interval mapping.

QTL	Pop	LG	Interval mapping						Genome-wide composite inteval Mapping					
			Position (cM)	Marker interval	LOD	Add	Dom	PVE (%)	Position (cM)	Marker interval	LOD	Add	Dom	PVE (%)
Anthracnose resistance	F_2_	13	67.7	sca82470	56.5	1.8	− 0.3	74.9	67.7	sca82470	61.93	1.73	0	73.06
	F_2:3_	13	67.7	sca82470	32.7	9.0	− 3.9	77.8	–	–	–	–	–	–
DTF	F_2_	4	nd	nd	nd	nd	nd	nd	81	sca409063~13840_95_80	4.74	0	1.72	0.4
		10	nd	nd	nd	nd	nd	nd	52.5	13561_55_79	5.08	− 1.5	0	0.6
		11	nd	nd	nd	nd	nd	nd	48	10497_69_66~sca172949	12.50	− 3.39	0	3.2
		23	68.1	sca43802	68.4	− 16.1	− 9.5	81.4	68.2	sca43802~13204_107_42	94.53	− 16.15	− 8.97	82.7
	F_2:3_	23	68.1	sca43802	15.0	− 4.6	− 0.7	30.8	–	–	–	–	–	–

*Pop*. population evaluated, *LG* linkage group, *PVE* proportion of phenotypic variance explained by QTL, *Add* additive effect, *Dom* dominance effect, *–* not analyzed in F_2:3_ family, *nd* not detected.

Despite the phenotypic performances showing clear evidence of a single major QTL for each trait, a further analysis was carried out to search for any minor segregating QTLs. By using a GCIM approach, no other minor segregating QTLs were detected in this F_2_ mapping population for anthracnose resistance, and the major QTL was detected (LOD = 61.9) and mapped to the same position as Interval Mapping (Fig. 4a, Table 3). However, for DTF three minor QTLs were detected and mapped on LG4 (LOD = 4.7), LG10 (LOD = 5.1) and LG11 (LOD = 12.5), together with the major QTL which was detected (LOD = 94.5) and mapped in the same position as Interval Mapping (Fig. 4b, Table 3).

In order to validate these major QTLs confirmed by three mapping methods, the marker sca82470 for anthracnose resistance and marker sca43802 for DTF, were used to validate each QTL (Table 3). These markers were then used to trace back the allelic constitution in both traits in each F_2:3_ family. The results showed that F_2_ plants had a good correlation between genotypic and the phenotypic values; it was noted that as expected for a single dominant gene, the LOD values, when based on mean performance and classes of F_2:3_ families, gave lower relative values to those based on individual F_2_ plant (Tables 2, 3). Thus, validating both QTL position on LG13 and LG23 in the *L. luteus* map.

### Candidate genes anthracnose resistance and DTF

By searching in the Lupin Genome Portal, nucleotide BLAST results on marker sca82470, which was highly associated with anthracnosis resistance in *L. luteus* in this study, produced a hit of 90.86% identity (E value = 0.00) to Scaffold_133 (Narrow-leafed lupin genome scaffold assembly v1.0) on pseudochromosome 11, 3376319–4744591 bp (Fig. 2b), where the *Lanr1* gene for anthracnose resistance has been localized^26^. The marker sequence of sca82470 predicted gene Lup005048. A further BLAST search on NCBI predicted *L. angustifolius* uncharacterized LOC 109360795 (LOC109360795) in this marker, with a 91.71% identity (E vale = 0.0).

DTF nucleotide BLAST result for marker sca43802 of *L. luteus* produced a 77.97% identity (E value = 0.00) to Scaffold_276_44 (Narrow-leafed lupin genome scaffold assembly v1.0) localized to pseudochromosome 10 from base positions 8,013,021 to 8,250,716 in *L. angustifolius* (Fig. 2d). The *LanFTc1* gene is localized to Scaffold_276_44 in *L. angustifolius*^26^. The marker sequence of sca43802 was homologous to *LanFTc1*, Lup015264. NCBI BLAST search in this marker predicted the presence of *L. angustifolius* protein TWIN SISTER of FT-like (LOC109357767), with a 93.13% identity (E value = 0.0). Out of three minor QTLs detected by GCIM, marker sca409063 flanking QTL on LG4, predicted the *L. angustifolius* floral homeotic protein AGAMOUS (LOC109345242) with a 97.91% identity (E value 2e-110) to *L. angustifolius*. With the marker 13561_55_79 flanking QTL on LG10, no significant similarity was found in NCBI database. The marker sca172949 flanking QTL on LG11, predicted a *L. angustifolius* CBS domain-containing protein CBSX1, chloroplastic-like (LOC109330470).

## Discussion

*Lupinus luteus* has high seed protein content with values 60% (DM) in dehulled seed have been achieved in south macro area of Chile^15^. This large area provides a favorable environment for lupins, with deep volcanic soil, deficient in phosphorus (P) but with abundant organic matter and high rainfall^53^, which helps explain the good performance of *L. luteus*. Lupins have the potential to mobilize scarcely available nutrients, in particular P and micronutrients, for themselves or subsequent crops. Their ability to symbiotically fix gaseous nitrogen is widely acknowledged as a factor contributing to soil improvement^4–6^. This natural adaptation for nutrient acquisition, not present in most major crops, is highly relevant for a sustainable agriculture, helping to face the global challenge of food security, with lower fertilizer and water footprints. However, elite germplasm of *L. luteus* is susceptible to anthracnose disease in high rainfall areas, meaning its yield can be dramatically reduced^19,54,55^. Even more, fluctuating temperatures, frost, day length, and other climate changes, means flowering time is another important trait. Breeding efforts toward combining optimal expression of these two key traits: anthracnose resistance and early flowering time/no vernalisation requirement, is indeed essential to allow expression of the yield potential in elite germplasm of this species. In *L. albus* this strategy has proved successful, despite the complexity, because of a low frequency of early flowering progenies and the apparent quantitative nature of anthracnose resistance in this species^20^. Flowering is a vital stage in plant development; it plays an important role in the initiation of grain setting and is highly sensitive to stresses^56^. The existence of DTF variability allows selection to maximize yield by optimizing plant phenology in different environments. It allows selection for better crop adaptation in different climatic conditions and different geographical regions^57^. Common flowering pathways and a number of highly conserved genes described across species have suggested a tight genetic control for this trait^58^.

Here, we report novel QTLs harboring a very early DTF gene from elite germplasm, and a single dominant gene *Lanr1*, from a wild accession, for anthracnose resistance in the *L. luteus* genome. These QTLs were mapped in a large F_2_ segregating population, with three QTL mapping approaches, consistent with previous studies of these traits in model legume plants and in the reference genome^30,59^. It is interesting that three significant minor QTLs associated with DTF, were detected only with the GCIM approach, but only one of these QTLs was harboring a candidate gene associated with flowering. The sequence of the marker sca409063 flanking this QTL, predicted the *L. angustifolius* Floral homeotic protein AGAMOUS (AG). The *AG* gene encoding a MADS-box transcription factor has been reported in plant flowering regulation in different species^60,61^. It is also relevant to point out that these significant minor QTLs were detected with GCIM-random methods, which is in agreement with Wen et al.^51^. Who have demonstrated the power of this method in the detection of QTL in F_2_ population.

Anthracnose resistance and DTF are a key combination of genes, and will allow better adaptation and thus fulfilment of the yield potential of *L. luteus* elite germplasm. This is even more relevant if it is noted that this species already has important natural adaptation for nutrient acquisition, allowing more sustainable agriculture. The mapping of *Lanr1* gene in *L. luteus*, allows interesting projections, since it was first identified in *L. angustifolius*, in which the cultivar Tanjil has been widely used for breeding anthracnose resistance in this species, because this single dominant gene had proved to show durable resistance^26,59^.

Our key result was the development of the genetic linkage map of *L. luteus* genome, which showed collinearity with the *L. angustifolius* reference map and syntenic genomic regions harboring major QTLs for these important traits. The advance of Next Generation Sequence technology and its wide application, together with genomic knowledge of the *L. angustifolius* reference genome, have greatly reduced the cost of nucleotide sequencing and facilitated our work. The work presented here may be the first example of using NextRAD markers to develop a genetic map in plants. The NextRAD markers were developed with a PCR step utilizing an oligo with a nine-nucleotide selective sequence to further reduce genome complexity. It is therefore expected that a higher density map of *L. luteus* can be directly prepared with more markers by simply removing or reducing this base-discrimination step. While the current map was suitable to identify candidate genes in two traits, a higher density map may be desirable for breeding future traits. We therefore conclude that NextRAD is a suitable approach for SNP discovery for genetic map development. The markers developed from the de novo assembly of *L. luteus* genome helped the comparative mapping studies with the reference map of *L. angustifolius*, by anchoring common markers in the maps of both lupin species, which confirmed LGs, map and syntenic regions found in this species.

Iqbal et al.^24^ recently described GBS for *L. luteus* whereby low-coverage sequencing data and imputation resulted in 948 selected SNP markers. The GBS markers from that study were combined with 2,006 DArT markers to produce a genetic map containing 40 LGs. Cytological studies of *L. luteus* support 2n = 52, or 26 LGs^11, 62^, thus the comparisons of this *L. luteus* map with the map generated in our study is complex. The map generated in this study represents 78.4% of the map length generated by Iqbal et al.^24^. Who covered a total length of 2,261 cM.

The mapping population used in this study had a wild male parent, containing valuable genetic variation for many traits. Interestingly, our results highlight that this cross with the wild accession also had the natural phenomenon of segregation distortion as observed in other species and crops^63^. According to the distribution of distorted markers along linkage groups of *L. luteus*, two types were identified: segregation distortion loci (SDL), i.e. loci widespread between chromosomes, with alleles in either parental class, and a segregation distortion region (SDR), i.e. markers showing highly significant distortion skewed in the same direction, clustered in a specific genomic region. SDRs, as found in LG17, with markers highly skewed toward the female parent, and in LG15, with markers highly skewed toward homozygosity i.e. to both parental types. An example of SDR is showed in Supplementary Table S6. This is the first reported distribution and direction of distorted markers in *L. luteus*, and shares the same features as other species and crops widely reported^64,65^. However, its genetic causation remains to be studied in *L. luteus*, as they are important when it is considered that the introduction of further wild accessions will be needed in order to reduce the genetic variation bottleneck apparent in this species.

Recently in soybean the effect of segregation distortion has been elucidated in mapping and QTL analysis, across diverse mapping populations and genetic backgrounds. Few chromosomes and clusters of markers generates complexity, and when distorted markers were included a more accurate map was obtained^65^. Interestingly, our results in *L. luteus* were very much in agreement with the results found in soybean, few chromosomes and genomic regions of *L. luteus* were mainly associated with segregation distortion. When these distorted markers were removed, the map generated was not entirely consistent with the map including all markers, that shared good collinearity and syntenic regions with the map and reference genome of *L. angustifolius*. In our study neither SDL or SDR were located in the genomic regions harboring QTLs for anthracnose resistance or DTF.

Since anthracnose resistance was studied in a large F_2_ mapping population and F_2:3_ families, in in vitro and field conditions, allowing the validation of methods and data. This supported the high LOD score found for this QTL, and identified a linked and fully co-segregating marker in the target syntenic region. High correlations between in vitro assay and field evaluation for anthracnose resistance have been reported previously^66,67^. Anthracnose resistance in several other legumes has been reported as being genetically control by a single dominant gene^59,68,69^, which is coherent with the single dominant gene mapped in this study. This also reflect the narrow range of pathogenic races of this fungus and follow the gene-for-gene model^70^. The assembly of homologous sequences with *L. angustifolius* in the syntenic region for anthracnose resistance QTL, suggested *Lanr1* as an orthologous candidate gene in the *L. luteus* genome (Figs. 2, 4). Moreover, BLAST results of genomic DNA sequences in the gene region of *L. angustifolius* chromosome 11 to EST sequences of *L. luteus* (SRA055806) produced a significant alignment, suggesting the gene encoded in this region was expressed^23^, supporting the QTL results of this study. In *L. angustifolius*, in a different genetic background, a fine map location of *Lanr1* showed complete conservation, allowing the proposal of an NLR gene as a likely candidate for *Lanr1*^26^. Flowering time was also evaluated in large F_2_ and F_2:3_ populations, in different environments and seasons, allowing the identification and validation, for the first time in this species, of a major QTL for this trait in the *L. luteus* genome. Like the *Lanr1* gene, it was also mapped in a syntenic and collinear genomic region of *L. angustifolius* reference genome. From previous studies in *L. angustifolius*, the *LanFTc1* gene has a large effect in terms of the regulation of DTF. FTc1 expression is increased with vernalisation in late genotypes of *L. angustifolius*^30^. In other legumes, such as *Glycine max*, *Pisum sativum* and *Medicago truncatula*, *FT* genes have been described as regulators of flowering time^71–73^.

The results of this study are in close agreement with previous studies in legumes; strongly suggesting that the QTL found in the *L. luteus* genome is harboring an orthologous *FTc1* gene, with highly probably of sharing similar functional effects as *L. angustifolius* and other legumes species.

The *L. luteus* map reported here, its collinearity and synteny of specific genomic regions allowed the discovery of important domestication traits. Considering these results, it is highly possible that *L. luteus* is also preserving collinearity with the reference legume genomes of *Arachis duranensis*, *Glycine max* and *Medicago truncatula*^25,74^.

Thus, the results of comparative mapping, synteny analysis, and presence of orthologous genes between *L. luteus* and the *L. angustifolius* reference genome, are suggesting that *L. luteus* LG13, LG23 and LG11 correspond to the pseudochromosomes: Chr11, Chr10 and Chr17, respectively.

The discoveries in this study provide strong validation of the synteny approach for transferring genomic knowledge from a model genome to a less well-resourced crop genome, with reduced time and economy of effort. It is proving that the sequence information in model genome plants, advanced molecular and functional information from species like *L. angustifolius*, are helping enormously to target and explore important genes for breeding improvement, especially under the pressure of sustainable production and food security.

## Supplementary information

Supplementary Figure S1.

Supplementary Figure S2.

Supplementary Figure S3.

Supplementary Figure S4.

Supplementary Table S1.

Supplementary Table S2.

Supplementary Table S3.

Supplementary Table S4.

Supplementary Table S5.

Supplementary Table S6.

Supplementary Table S7.

Supplementary Table S8.

Supplementary Table S9.
