# Supplementary material for: Genetic and comparative mapping of Lupinus luteus L. highlight syntenic regions with major orthologous genes controlling anthracnose resistance and flowering time
Source: Sci Rep. 2020 Nov 5;10:19174. doi: 10.1038/s41598-020-76197-w (PMC7645761; doi:10.1038/s41598-020-76197-w)
Supplement: Supplementary file 2 — Supplementary Figure S2. [file 41598_2020_76197_MOESM2_ESM.docx]

**Genetic and comparative mapping of *Lupinus luteus* L. highlight syntenic regions with major orthologous genes controlling anthracnose resistance and flowering time**

Nicole Lichtin^1^, Haroldo Salvo-Garrido^1^, Bradley Till^1^, Peter DS Caligari ^1^, Annally Rupayan^1^, Fernando Westermeyer^1^ and Marcos Olivos ^1^

Author affiliations:

^1^ CGNA (Agriaquaculture Nutritional Genomic Center), Las Heras 350, Temuco, Chile

*Corresponding author: Haroldo Salvo-Garrido; haroldo.salvo@cgna.cl


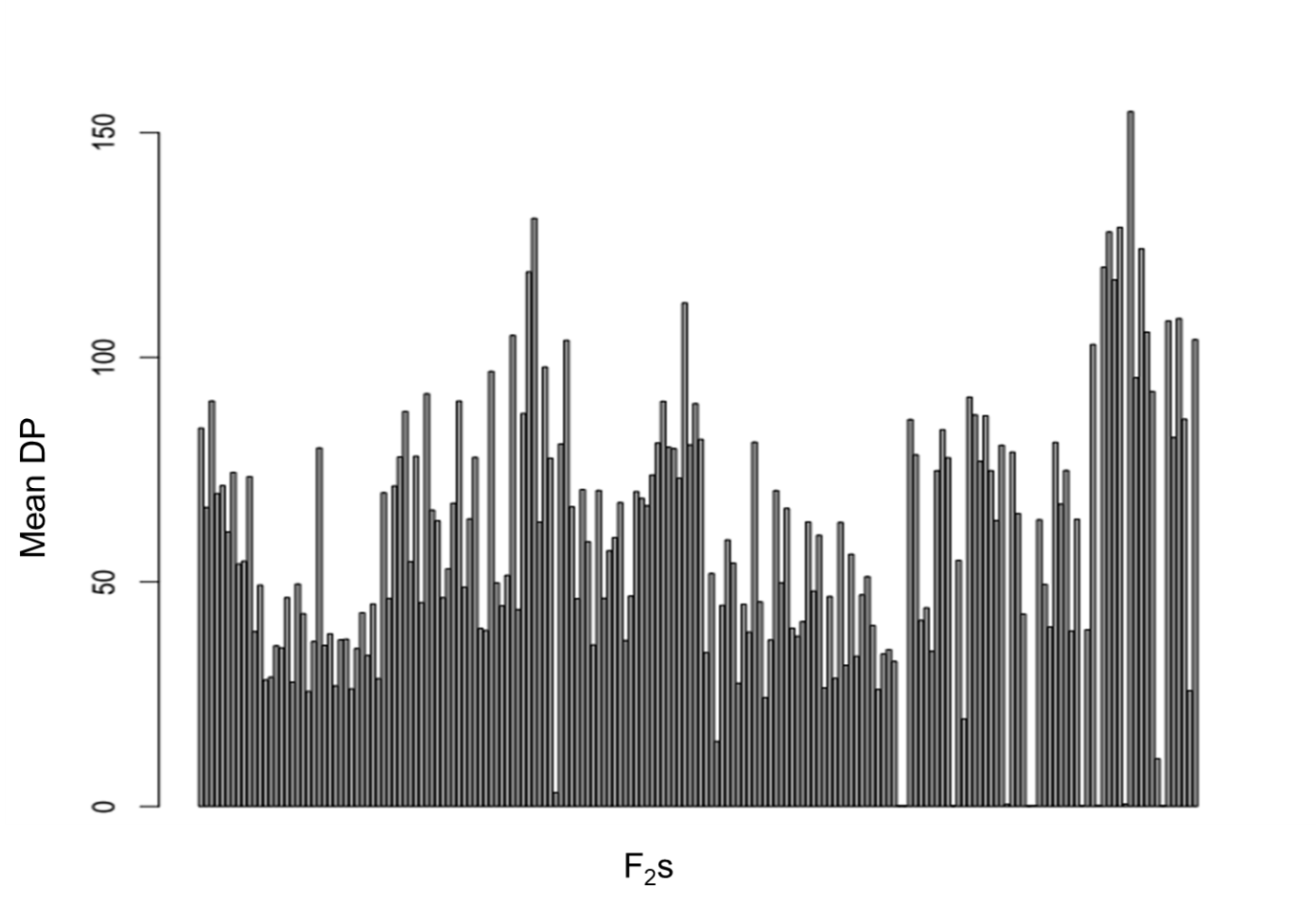


**Supplementary Figure S2.** Mean coverage in F_2_ individuals for all NextRAD markers. DP: Sequencing depth

Software used: The barplot function in R. R Core Team (2019). R: A Language and Environment for Statistical Computing, R Foundation for Statistical Computing, Vienna, Austria, https://www.R-project.org
