# Supplementary material for: Genetic and comparative mapping of Lupinus luteus L. highlight syntenic regions with major orthologous genes controlling anthracnose resistance and flowering time
Source: Sci Rep. 2020 Nov 5;10:19174. doi: 10.1038/s41598-020-76197-w (PMC7645761; doi:10.1038/s41598-020-76197-w)
Supplement: Supplementary file 3 — Supplementary Figure S3. [file 41598_2020_76197_MOESM3_ESM.docx]

**Genetic and comparative mapping of *Lupinus luteus* L. highlight syntenic regions with major orthologous genes controlling anthracnose resistance and flowering time**

Nicole Lichtin^1^, Haroldo Salvo-Garrido^1^, Bradley Till^1^, Peter DS Caligari ^1^, Annally Rupayan^1^, Fernando Westermeyer^1^ and Marcos Olivos ^1^

Author affiliations:

^1^ CGNA (Agriaquaculture Nutritional Genomic Center), Las Heras 350, Temuco, Chile

*Corresponding author: Haroldo Salvo-Garrido; haroldo.salvo@cgna.cl


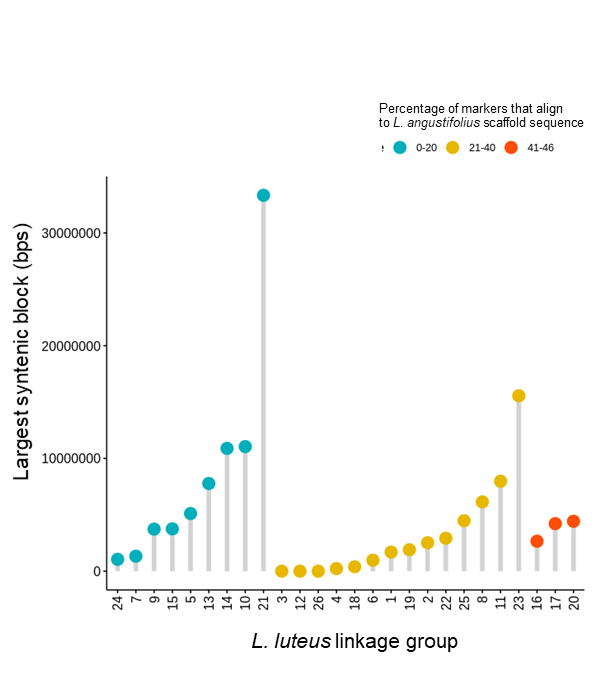


**Supplementary Figure S3.** Lollipop chart showing the largest syntenic blocks between *L. luteus* linkage groups and the *L. angustifolius* genome, based on BLAST alignment. The *L. luteus* linkage groups are divided and sorted into three sub-groupings, based on the percentage of markers in the linage group that align to *L. angustifolius* scaffold sequence. Blue dots represent groups with 0-20% of markers aligning scaffold sequence, yellow 21-40% and red, 41-46%.

Software used: R package ggplot2. Wickham H (2016). ggplot2: Elegant Graphics for Data Analysis. Springer-Verlag New York. ISBN 978-3-319-24277-4, [https://ggplot2.tidyverse.org](https://ggplot2.tidyverse.org/). R Core Team (2019). R: A Language and Environment for Statistical Computing, R Foundation for Statistical Computing, Vienna, Austria, https://www.R-project.org.
