# Supplementary material for: Genetic and comparative mapping of Lupinus luteus L. highlight syntenic regions with major orthologous genes controlling anthracnose resistance and flowering time
Source: Sci Rep. 2020 Nov 5;10:19174. doi: 10.1038/s41598-020-76197-w (PMC7645761; doi:10.1038/s41598-020-76197-w)
Supplement: Supplementary file 4 — Supplementary Figure S4. [file 41598_2020_76197_MOESM4_ESM.docx]

**Genetic and comparative mapping of *Lupinus luteus* L. highlight syntenic regions with major orthologous genes controlling anthracnose resistance and flowering time**

Nicole Lichtin^1^, Haroldo Salvo-Garrido^1^, Bradley Till^1^, Peter DS Caligari ^1^, Annally Rupayan^1^, Fernando Westermeyer^1^ and Marcos Olivos ^1^

Author affiliations:

^1^ CGNA (Agriaquaculture Nutritional Genomic Center), Las Heras 350, Temuco, Chile

*Corresponding author: Salvo-Garrido Haroldo; haroldo.salvo@cgna.cl


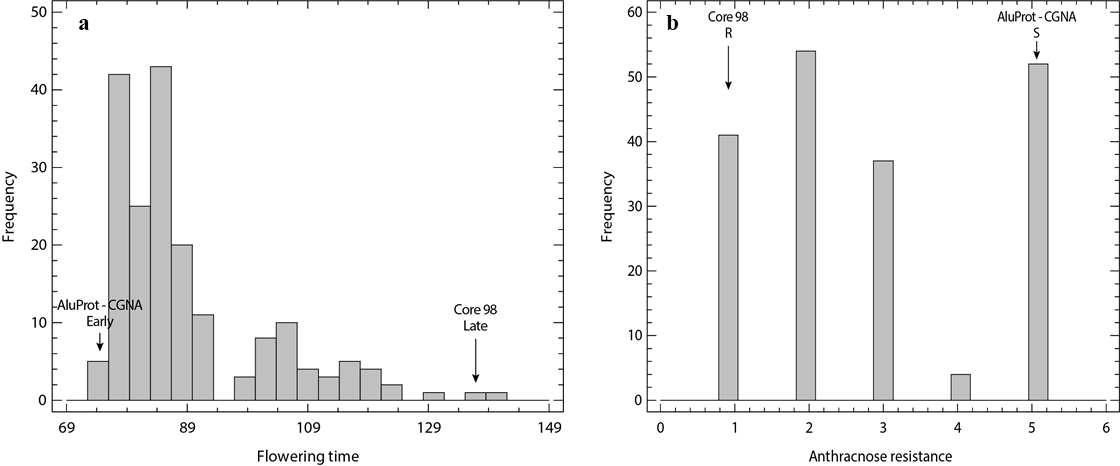


**Supplementary Figure S4.** Frequency distribution of flowering time (DTF) (a) and anthracnose resistance (b) in the F_2_ mapping population, from a cross of wild *L. luteus* accession Core 98 (late flowering, resistance (R)) and cultivar Alu*Prot*-CGNA (early flowering, susceptible (S)). Arrows indicates mean performance of parents.

Software used: SAS, Excel
